# Mapping the microRNA landscape in the older adult brain and its genetic contribution to neuropsychiatric conditions

**DOI:** 10.1038/s43587-024-00778-x

**Published:** 2024-12-06

**Authors:** Selina M. Vattathil, Ekaterina S. Gerasimov, Se Min Canon, Adriana Lori, Sarah Sze Min Tan, Paul J. Kim, Yue Liu, Eric C. Lai, David A. Bennett, Thomas S. Wingo, Aliza P. Wingo

**Affiliations:** 1 Department of Neurology, University of California, Davis, Sacramento, CA USA; 2 Department of Psychiatry, University of California, Davis, Sacramento, CA USA; 3 Department of Neurology, Emory University School of Medicine, Atlanta, GA USA; 4 Department of Psychiatry, Emory University School of Medicine, Atlanta, GA USA; 5 Developmental Biology Program, Sloan Kettering Institute, New York, NY USA; 6 Rush Alzheimer’s Disease Center, Rush University Medical Center, Chicago, IL USA; 7 Alzheimer’s Disease Research Center, University of California, Davis, Sacramento, CA USA; 8 Veterans Affairs Northern California Health Care System, Sacramento, CA USA

**Keywords:** Gene expression, Gene regulation, Brain, Ageing

## Abstract

MicroRNAs (miRNAs) play a crucial role in regulating gene expression and influence many biological processes. Despite their importance, understanding of how genetic variation affects miRNA expression in the brain and how this relates to brain disorders remains limited. Here we investigated these questions by identifying microRNA expression quantitative trait loci (miR-QTLs), or genetic variants associated with brain miRNA levels, using genome-wide small RNA sequencing profiles from dorsolateral prefrontal cortex samples of 604 older adult donors of European ancestry. Here we show that nearly half (224 of 470) of the analyzed miRNAs have associated miR-QTLs, many of which fall in regulatory regions such as brain promoters and enhancers. We also demonstrate that intragenic miRNAs often have genetic regulation independent from their host genes. Furthermore, by integrating our findings with 16 genome-wide association studies of psychiatric and neurodegenerative disorders, we identified miRNAs that likely contribute to bipolar disorder, depression, schizophrenia and Parkinson’s disease. These findings advance understanding of the genetic regulation of miRNAs and their role in brain health and disease.

## Abstract (web summary)

Using small RNA sequencing data from aged human brain tissue, Vattathil, Gerasimov et al. uncovered genetic variants that influence microRNA (miRNA) expression and, by integrating genome-wide association study data, identified miRNAs linked to the etiology of psychiatric and neurodegenerative disorders.

## Main

MicroRNAs (miRNAs) are small non-coding RNAs that suppress gene expression by binding complementary mRNA sequences and causing either transcript degradation or translation repression. miRNAs are typically produced through multi-step post-transcriptional processing that begins with cleavage of the primary miRNA transcript (hundreds or thousands of nucleotides long) into a hairpin-shaped precursor miRNA (pre-miRNA), which is transported into the cytoplasm and then cleaved into approximately 22-nucleotide (nt) mature miRNAs^1^. Each pre-miRNA can produce up to two mature miRNAs, one from each arm of the hairpin. Each miRNA can influence the expression of potentially hundreds of genes and thereby exert a broad influence on gene expression^2^, which makes them relevant for understanding the complex networks of interacting genes involved in most human disease.

Although miRNAs have a well-described role in gene regulation, less is known about how they themselves are genetically regulated. In contrast, comparatively more is known about how genetic variants are associated with variation in transcript and protein expression—so called quantitative trait loci (QTL) mapping. This has led to important insights into human conditions, especially by integrating QTLs with genome-wide association study (GWAS) results^3–6^. Thus, we hypothesize that miRNA QTLs (miR-QTLs) ought to provide similar insights.

Characterizing miR-QTLs has several additional benefits. First, because miRNAs regulate genes that may be distally located in the genome, miR-QTLs could be used to reduce the multiple testing burden for finding *trans*-protein QTLs (pQTLs). Second, miR-QTLs could be used to test whether miRNAs mediate protein expression when both the miRNA and protein are under genetic control, which would complement existing miRNA target prediction approaches. Together, these results could provide a more complete picture of gene expression regulation.

Given their small size, miRNAs identified from next-generation sequencing data are prone to false annotation if appropriate criteria are not applied^7,8^. Coupled with the fact that miRNAs may be cell type-specific^9^, the precise complement of human miRNAs remains a subject of debate^10,11^.

**Fig. 1:**
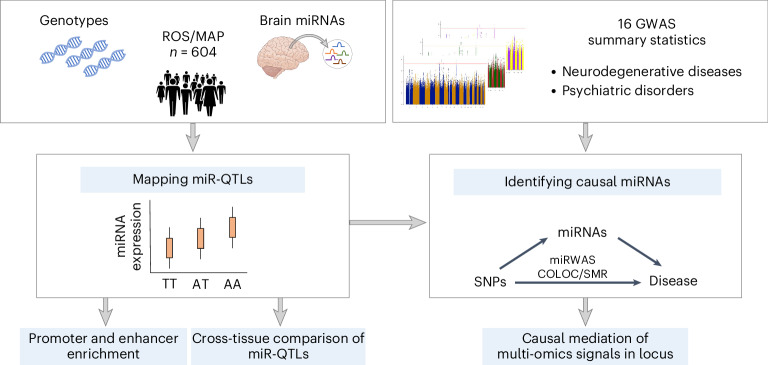
Main analyses. The miRNA data generated in this study were compared and integrated with multiple existing datasets and resources.

In the present study, we first cataloged miR-QTLs using small RNA sequencing from adult human dorsolateral prefrontal cortex (dlPFC) (Fig. 1). Given the current uncertainty about the true miRNA complement, we conducted parallel miR-QTL mapping using miRNAs from two miRNA databases that have different inclusion criteria. Next, we leveraged the high-confidence miR-QTLs to address questions about gene regulation, such as whether miRNAs are regulated independently of host genes; whether miR-QTLs are enriched in promoters or enhancers; whether miR-QTLs can be used to detect pQTLs and support predicted miRNA targets; and whether miR-QTLs are tissue-specific. Finally, we applied multiple complementary methods for integrating the miR-QTLs with GWAS signals from 16 neurodegenerative and psychiatric conditions to identify miRNAs that mediate the effect of genetic variation on the brain-related disorders (Fig. 1). Altogether, we uncovered miRNAs involved in brain illnesses, characterized features of miRNA regulation, and provide a human brain miR-QTL resource for future study.

## Results

### miR-QTL mapping

To catalog miR-QTLs, we performed miRNA sequencing on dlPFC samples from participants of the Religious Orders Study/Rush Memory and Aging Project (ROS/MAP) and combined these data with available matched genotype data. miRNA and genotype data from 604 individuals were available for analysis after quality control filtering on the combined dataset. All participants were over 70 years of age at the time of death, and the median age of death was 90 years. Approximately 70% (432 of 604) of the participants were female. All individuals in the analysis were of European genetic ancestry. The median miRNA sequencing depth per sample was 27.3 million reads (range, 13.2–37.4 million). Additional sample and data characteristics are summarized in Supplementary Table 1.

We chose to independently map miRNA sequencing reads to two reference miRNA databases: miRBase^12^ and MirGeneDB^13,14^. The rationale for this was that, although miRBase is a widely used database in the research community, it has been noted for potentially including a substantial number of falsely annotated miRNAs^8,15–17^. On the other hand, MirGeneDB is a manually curated miRNA database with reported low rate of false annotations and high completeness for human miRNAs^13,14^.

**Extended Data Fig. 1:**
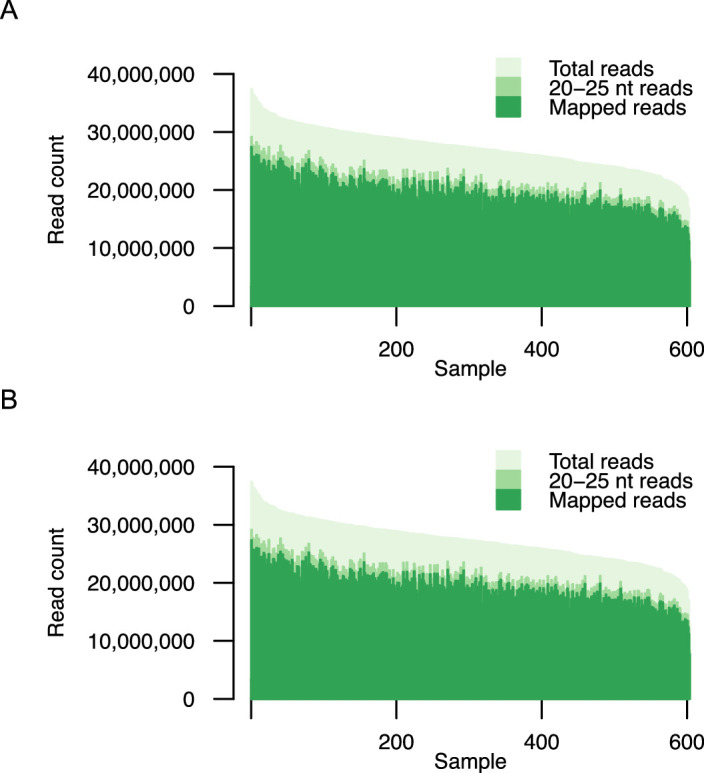
Small RNA sequencing depth and mapped read counts. The count of total reads, reads with length 20-25 nucleotides, and mapped reads are plotted in light green, medium green, and dark green, respectively. Samples are sorted by decreasing total read count. **a**) reads mapped to miRBase miRNAs; **b**) reads mapped to MirGeneDB miRNAs.

Using miRBase to map miRNA reads resulted in a median of 18.8 million mapped reads per sample (range, 7.7–27.4 million) (Extended Data Fig. 1). After filtering based on our abundance criteria (≥1 reads per million (RPM) for ≥50% of samples), 624 autosomal miRNAs were available for miR-QTL analysis (Supplementary Tables 2 and 3). Considering single-nucleotide polymorphisms (SNPs) within 500 kilobases (kb) of the precursor sequences, we tested 1,315,257 SNP–miRNA pairs and observed miR-QTLs for 326 miRNAs at false discovery rate (FDR) < 1% (unadjusted *P* < 2.98 × 10^−4^). Hereafter, miRNAs with at least one miR-QTL are referred to as eMiRs. Because we analyzed a dense set of genotypes, many SNPs are in high linkage disequilibrium (LD). To identify a reduced set of SNPs, we applied LD-based clumping to the miR-QTL results to identify ‘index’ miR-QTLs for each eMiR, followed by conditional analysis to identify conditionally independent miR-QTLs for each eMiR. Of the 326 eMiRs, 71% had one conditionally independent miR-QTL, 22% had two, and 7% had three, four, or five.

**Fig. 2:**
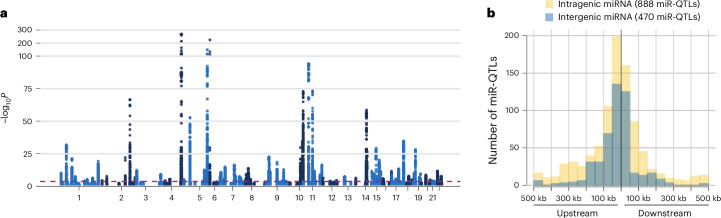
Summary of miR-QTL results. **a**, Manhattan plot of miR-QTL results. Each point represents a SNP–miRNA association. The *y* axis represents unadjusted *P* values. The pink dashed line indicates *P* value threshold corresponding to FDR of 1%. The *y* axis scale is condensed for −log_10_*P* ≥ 100. **b**, Distribution of miR-QTL position relative to the miRNA precursor for 1,358 index miR-QTLs. The miR-QTLs were stratified by whether the associated miRNA is intragenic (yellow) or intergenic (blue), and the two histograms are overlaid. The plot excludes 21 index miR-QTLs that had ambiguous relative position (because they were between or among precursors), were within the precursor, or were associated with miRNAs that could not be classified as intergenic or intragenic.

Using MirGeneDB to map miRNA reads resulted in a median of 18.7 million mapped reads per sample (range, 7.7–27.3 million). The list of tested MirGeneDB miRNAs and RPM statistics are in Supplementary Tables 4 and 5. Using the MirGeneDB miRNA set at FDR < 1% (unadjusted *P* < 2.34 × 10^−4^), we observed miR-QTLs for 224 of 470 tested miRNAs (Fig. 2a). The distribution of the conditionally independent miR-QTLs per eMiR is similar to what we observed with miRBase. Specifically, of the 224 eMiRs, 75% had one conditionally independent miR-QTL, 18% had two, and approximately 6% had three or four.

Notably, MirGeneDB miRNAs captured over 99.7% of the reads that mapped to the miRBase miRNAs, indicating that the miRNAs unique to miRBase are sparsely observed in our sequence data. Furthermore, the tested miRNAs in the MirGeneDB set included 75% (470 of 624) of the tested miRNAs from the miRBase set. This substantial overlap in the tested miRNAs may be surprising at first given that miRBase lists several times more miRNAs than MirGeneDB. The simplest explanation is that miRBase-specific miRNAs are rarely detected in our data at or above our minimum required abundance threshold. Consistent with this, the tested miRNAs specific to miRBase tended to have lower expression than those present in both databases (median RPM of 3 versus 51, two-sided Wilcoxon rank-sum test *P* = 7.8 × 10^−31^).

The mapped read counts and miR-QTL summary statistics for both the miRBase and MirGeneDB miRNA sets are made available for researchers (see ‘Data availability’ section). For the downstream analyses in the rest of the paper, we opted to use the miR-QTLs from the MirGeneDB miRNAs to focus on miRNAs that have the same high confidence support.

### miR-QTL characteristics

The median percent of miRNA expression variance explained by each conditionally independent miR-QTL was 4.1% (interquartile range (IQR) 2.7–7.8%), and 18 eMiRs have a miR-QTL that explains more than 20% of their expression variance. These values reflect not only the true effect size distribution of miR-QTLs but also the detection power in this study. We expect to have captured the strongest miR-QTLs; larger studies would be expected to identify additional miR-QTLs with smaller effect sizes.

Two features of miRNA biology can create correlation in mature miRNA abundances. First, each precursor potentially encodes two mature miRNAs, from the −3p and −5p hairpin arms. Second, miRNA genes are often clustered in the genome and may be transcribed together. Given these features, we examined miR-QTL sharing among clustered miRNAs. We can define a cluster as miRNA genes separated by fewer than 10 kb; others are standalone genes. Using these definitions, the 224 eMiRs originate from 181 precursors at 135 distinct loci (clusters or standalone genes). We observed moderate correlation between miRNAs from the same precursor (median Pearson correlation, 0.50; IQR 0.25–0.68) or cluster (median Pearson correlation, 0.43; IQR 0.27–0.70). Predictably, miR-QTLs were sometimes shared by miRNAs from the same precursor or cluster, but 67% of SNPs tested for both the −3p and −5p miRNAs from the same precursor were associated with only one.

We characterized the position of the 1,384 index miR-QTLs relative to their precursor(s). Three index miR-QTLs were located within the miRNA precursor sequence, including one located within the mature miRNA (Supplementary Table 7). Approximately 9% of tested miRNAs mapped to multiple precursors; for one such miRNA, the two index miR-QTLs were located between precursors in a cluster, making it difficult to categorize them. Among the remaining miRNAs, miR-QTLs were more often upstream than downstream of the gene (809 versus 570, two-sided binomial test *P* = 1.32 × 10^−10^) (Fig. 2b and Supplementary Table 8). As expected, miR-QTLs tended to be near the transcription start site (TSS), with distance significantly correlated with *P* value (Spearman rho, 0.23; *P* = 1.14 × 10^−17^). Additionally, miR-QTLs for intragenic miRNAs were farther from the miRNA precursor than those for intergenic miRNAs (two-sided Wilcoxon rank-sum *P* = 6.31 × 10^−12^).

### Extent of co-localization with host gene eQTLs and pQTLs

Approximately 60% (135) of the eMiRs originate from intragenic miRNA precursors. Previous work showed that intragenic miRNAs are sometimes co-expressed with their host genes^18,19^. Consistent with this, we found that intragenic miRNA abundance was more likely to be positively correlated with host gene transcripts than expected by chance, using matched dlPFC miRNA and transcriptomic data from 297 participants (two-sided Wilcoxon rank-sum *P* = 5.6 × 10^−13^). To explore genetic co-regulation, we used coloc^20^, a Bayesian co-localization method, to compare miR-QTLs and eQTLs from 621 ROS/MAP participants^21^. Among 106 miRNA–host pairs (103 mature miRNAs from 77 precursors in 71 host genes) for which the miRNA had miR-QTLs and the host gene had eQTLs, we found evidence of co-localization (coloc PP.H4 > 0.5) for 17 pairs and none for 89 pairs (Supplementary Table 9). This suggests that miRNA expression and host gene expression are usually regulated independently, or that post-transcriptional regulation breaks their association. In any case, the findings show that for most intragenic miRNAs, the miR-QTL signal is not explained by host gene eQTLs, and miR-QTLs provide information that is complementary to that from eQTLs.

Despite the tendency for positive correlation between miRNA and host gene transcript abundance, two of the miRNA–host pairs with co-localized QTLs showed significant negative correlation. For one pair, miR-149-5p and its host gene *GPC1*, previous work showed that miR-149-5p targets *GPC1* in endothelial cells, has its own intronic promoter, and is activated independently of the host gene^22^. Our results, combined with these findings, suggest that the *GPC1* eQTL signal may be driven by the miR-QTL effect on miR-149-5p and demonstrate that considering miR-QTLs can reveal the mechanism of action of host gene eQTLs. For the second pair, miR-1908-5p and host gene *FADS1*, we found no experimental validation of the interaction, but our Mendelian randomization analysis (see below) supports this interaction.

We also tested co-localization between miR-QTLs and host gene pQTLs using proteomics data for 52 of the 106 miRNA–gene pairs (Supplementary Table 10). Co-localization or lack thereof was consistent at the eQTL and pQTL level for 37 pairs, whereas eight pairs showed co-localization only at the eQTL level and five only at the pQTL level. Previous studies found weak correlation between transcript and protein abundance^23–26^, so the discordance may be explained by factors such as post-transcriptional regulation of the miRNA or host gene product.

### Association with abundance of downstream target proteins

miRNAs are of interest because of their potential role in regulating target gene abundance. Although regulatory networks are complex and miRNAs are unlikely to be the sole regulatory mechanism for any given gene, we hypothesized that some eMiRs would have miRNA-QTLs significantly associated with the protein expression of their target genes. Specifically, we expected to find a small number of miR-QTLs that are also pQTLs (*cis* or *trans*) for these genes. To test this, we obtained predicted target genes for each eMiR from TargetScan 7.2 and examined whether miR-QTLs were pQTLs for predicted targets using genotype and dlPFC proteomic data from up to 716 participants of ROS/MAP and the Arizona Study of Aging, 511 of whom were part of our miR-QTL dataset.

There were 206 eMiRs for which predicted downstream target genes were profiled in our dlPFC proteomics dataset. The median number of predicted targets per miRNA was 249 (IQR 165–364). For all except 44 of approximately 59,000 miRNA–protein pairs, the protein is *trans* (>500 kb away or on a different chromosome) to the miRNA, meaning that we were mostly conducting *trans*-pQTL analysis. Although recent work suggests that *trans*-QTLs collectively contribute substantially to complex trait heritability, individual *trans*-QTLs have small effects and are difficult to detect at smaller sample sizes^27^, especially when conducting naive genome-wide searches. By focusing pQTL tests on SNPs already identified as miR-QTLs for predicted targets, we reduce the multiple testing burden.

Of the approximately 59,000 miRNA-predicted target pairs (involving 206 miRNAs and 7,999 proteins), we found miR-QTL and pQTL sharing for 11 pairs (Supplementary Table 11). These involved 10 mature miRNAs from nine precursors and 10 unique target genes. One miRNA (miR-1307-5p) had pQTLs for two genes (LYPLA2 and PPFIA3), and both miR-1307-3p and miR-1307-5p had pQTLs for PPFIA3. For eight of the 11 pairs, the QTL SNPs were *trans* to the target gene. Although the QTL sharing that we detected is modest, it aligns with the expected small effect sizes for *trans*-pQTLs.

We further characterized the relationship among miR-QTL, miRNA expression and protein expression for these 11 miRNA–target pairs using summary data-based Mendelian randomization (SMR) and HEIDI tests^28^. These Mendelian randomization–based methods distinguish instances of causality (SNP effects on protein are mediated by miRNA) and pleiotropy (SNP affects both miRNA and protein directly) from linkage (distinct SNPs are each associated with miRNA or protein but are in LD). Five of the 11 pairs showed significant SMR results after Bonferroni correction for the 11 SMR tests (*P* < 4.54 × 10^−3^), and four had HEIDI *P* values suggesting causality or pleiotropy (Table 1 and Supplementary Table 12). We can consider the relative locations of the miRNA and target gene and the QTL effect directions to further interpret the relationship among the SNP, miRNA and protein. One pair, the intragenic miRNA miR-185-3p and its host gene, *TANGO2*, suggest a pleiotropic effect, as the top SMR SNP is located upstream of the host gene and is associated with miRNA and protein abundance in the same direction. The other three pairs involve miRNAs and target genes located on separate chromosomes (Table 1). Causality seems more likely than pleiotropy for these pairs given the low frequency of *trans*-acting regulatory regions and that the top SMR SNP is associated with miRNA and protein abundance in opposite directions.

**Table 1:** miRNA and target gene pairs with evidence from SMR and HEIDI that genetically regulated miRNA abundance is associated with target protein abundance

miRNA	miRNA median RPM	Target protein	miRNA position relative to target protein	miR-QTL effect direction	pQTL effect direction	SMR *P* value	HEIDI *P* value
miR-1307-5p	48	LYPLA2	*trans*	Down	Up	2.14 × 10^−10^	0.64
miR-1307-3p	475	PPFIA3	*trans*	Down	Up	9.25 × 10^−8^	0.36
miR-1307-5p	48	PPFIA3	*trans*	Down	Up	1.36 × 10^−7^	0.42
miR-185-3p	34	TANGO2	Intronic, same strand	Up	Up	1.46 × 10^−7^	0.39

The SMR and HEIDI *P* values were estimated using SMR^28^.

**Extended Data Fig. 2:**
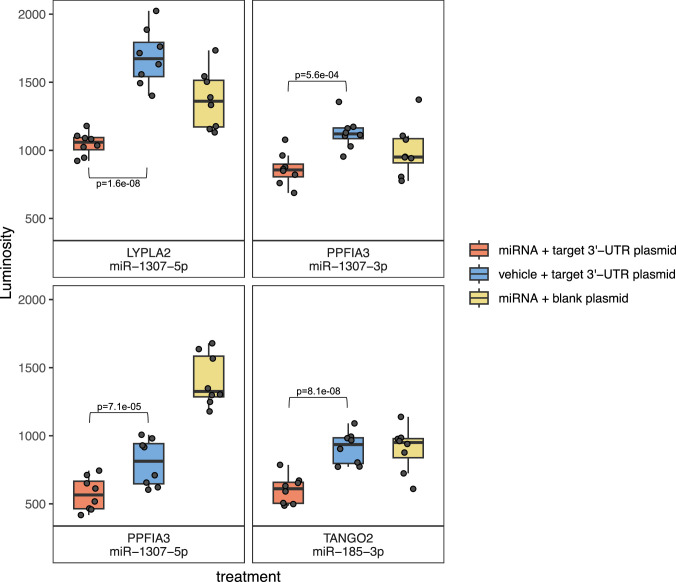
Validation of miRNA-target interaction using luciferase assays. Each panel corresponds to one of the four miRNA-target pairs with significant SMR/HEIDI results. For all pairs, luciferase expression was significantly lower in assays transfected with the miRNA mimic than in assays transfected with vehicle only (red v. blue boxplots, using linear regression with adjustment for plate effect), providing validation for miRNA-target interaction. The assays involving blank plasmid (yellow boxplots) were performed to verify that transfection with miRNA mimic did not interfere with luciferase readings. Each assay was performed in eight replicates. Center line indicates median, hinges indicate 25^th^ and 75^th^ percentile, and whiskers indicate 1.5 × the interquartile range. Individual data points have been overlaid over the boxplots.

The four miRNA–target pairs are not found in TarBase^29^, a database of experimentally validated miRNA–target gene interactions. To experimentally validate the potential interactions, we conducted luciferase assays in HEK293T cells using gene 3′ untranslated region (UTR) reporter constructs and miRNA mimics. The results support interaction for all four pairs (Extended Data Fig. 2), adding plausibility to the causal relationships inferred from our bioinformatic analyses.

TargetScan predicts targets only in the 3′ UTR, but targets can occur in other transcript regions and prediction tools use varying criteria. To expand our analysis, we collected miRNA–target interactions predicted by RNA22 (ref. ^30^) version 2 and repeated the pQTL and SMR/HEIDI tests. This increased the number of predicted interactions from approximately 59,000 to approximately 184,000, identifying two additional miRNA–target pairs with SMR/HEIDI support for pleiotropy or causality (Supplementary Table 12). In both cases, the top SMR SNP was associated with miRNA and protein abundance in the same direction, which does not suggest a causal role for the miRNA. Overall, the three *trans* miRNA–target pairs listed in Table 1 remain the strongest candidates for miRNAs that mediate the *trans*-pQTL effect on their predicted target genes.

### Enrichment in gene promoters and enhancers

We hypothesized that miR-QTLs were enriched in regulatory regions. To investigate this, we intersected the miR-QTLs with cell type-specific promoters and enhancers identified for neurons, astrocytes, oligodendrocytes and microglia by Nott et al.^31^.

**Fig. 3:**
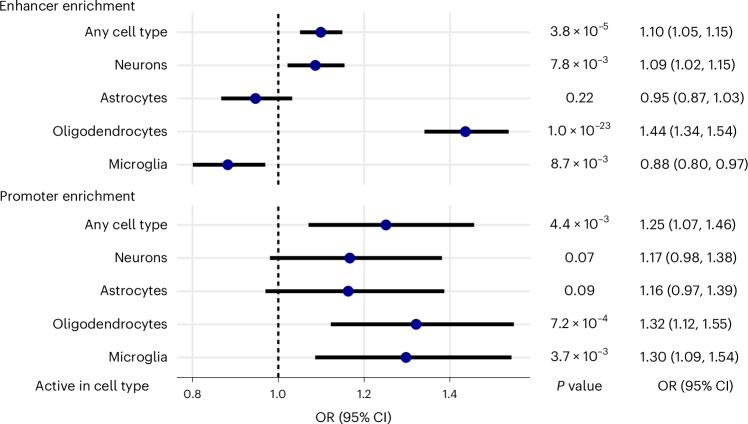
miR-QTL enrichment in brain enhancers and promoters. Enrichment was evaluated with the Mantel–Haenszel test using 37,348 SNPs within 50 kb of miRNAs for promoters and 357,395 SNPs within 500 kb of miRNAs for enhancers. Data are presented as OR ± 95% CI. *P* values were not adjusted for multiple testing.

We found that miR-QTLs were enriched (*P* < 0.05) in brain promoters combined across all four cell types (Fig. 3). The Nott et al.^31^ study indicated that promoters were largely shared among these cell types^31^. Supporting this, of the 1,272 miR-QTLs that overlap promoters, 61% overlap promoters in all four cell types. When testing promoter enrichment in each cell type separately, we did not find statistically significant enrichment for astrocyte and neurons; however, a consistent trend was observed across cell types (Fig. 3).

We found that miR-QTLs were enriched in the union set of enhancers across all four cell types (Fig. 3). Unlike promoters, enhancer activity was largely cell type specific. Examining enhancers for each cell type separately, miR-QTLs were enriched in oligodendrocytes and neuron enhancers but not astrocytes; conversely, miR-QTLs were depleted in microglia enhancers (Fig. 3). We also tested miR-QTL enrichment in the reference enhancer list from PsychEncode, derived from bulk prefrontal cortex tissue^32^, and found significant enrichment (Mantel–Haenszel common odds ratio (OR) = 1.32; 95% confidence interval (CI): [1.20, 1.45]; *P* = 1.3 × 10^−8^). This OR is within the range observed for cell-type-specific enhancer enrichments. We hypothesize that the depletion in microglia may be an artifact caused by the small proportion of microglia in the cells used for our bulk miR-QTL analysis. Consequently, genetic variants affecting miRNA abundance specifically in microglia may be less detectable compared with those from more prevalent cell types. This bias, combined with the cell type-specific nature of enhancers, could explain the lower rate of miR-QTLs in microglia enhancers relative to non-microglia enhancers.

### Comparison with other tissues

We compared our miR-QTL results with two recent studies conducted in different human tissues. The first study used qRT–PCR to measure miRNA expression in whole blood from up to 5,239 participants of the Framingham Heart Study, identifying *cis*-miR-QTLs (within 1 megabase (Mb) of the mature miRNA) for 76 of 280 miRNAs^33^. The second study analyzed small RNA sequencing from 212 fetal brain neocortex samples, uncovering *cis*-miR-QTLs (within 1 Mb of the miRNA precursor) for 65 of 811 mature miRNAs at their stringent threshold^34^.

**Extended Data Fig. 3:**
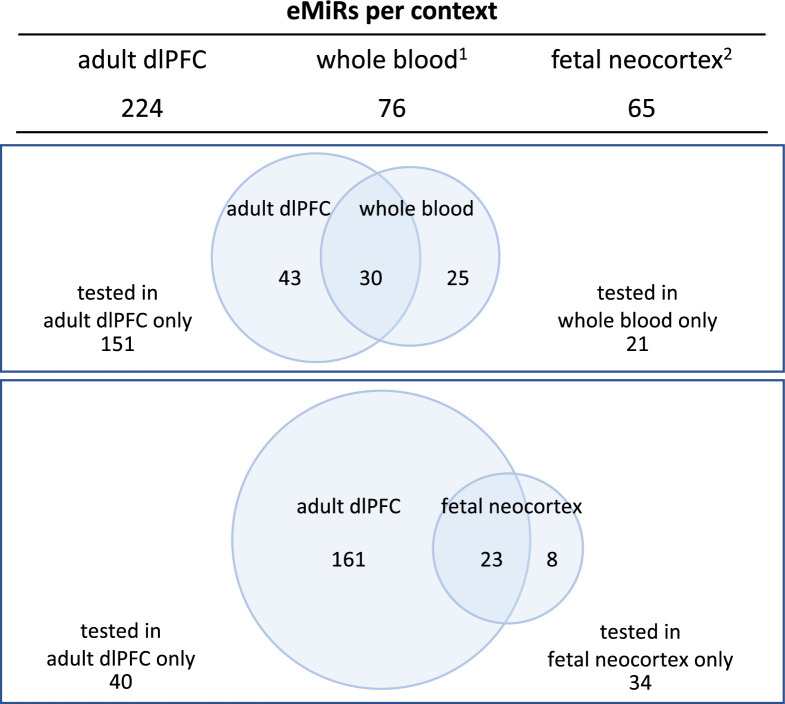
Comparison of eMiRs (miRNAs with miR-QTLs) across contexts. The Venn diagrams summarize the number of shared and unique eMiRs between this adult dlPFC study and published eMiRs from whole blood and fetal (midgestation) neocortex. Seven miRNAs (miR-130b-3p/-5p, miR-204-5p, miR-31-5p, miR-323a-3p, miR-339-3p, miR-411-3p) were eMiRs in all three studies. ^1^https://doi.org/10.1038/ncomms7601; ^2^ https://doi.org/10.7554/eLife.79488

To assess the rate of eMiR sharing between our study and the other two studies, we compared eMiR counts based on the miRNAs tested in each study (Extended Data Fig. 3). We first compared our eMiRs to those discovered in whole blood. Of the 224 eMiRs that we identified, 73 were tested in whole blood, and 41% of those (30 of 73) were identified as eMiRs in that context. Conversely, of the 76 eMiRs identified in whole blood, 55 were tested in our study, and 55% (30 of 55) were identified as eMiRs (Extended Data Fig. 3). A chi-squared test on the 173 miRNAs tested in both studies indicates that the number of shared eMiRs is higher than expected by chance, *X*^2^(1, *n* = 173) = 4.3, *P* = 0.038.

It is crucial to note that the detected miR-QTLs reflect not only differences in miRNA genetic regulation across contexts but also the sensitivity and specificity of each study. In the comparison to the fetal neocortex results, only 13% (23 of 184) of the eMiRs that we identified were also identified as eMiRs in the fetal neocortex study; however, 74% (23 of 31) of the fetal eMiRs were evident in our analysis (Extended Data Fig. 3). These results suggest that differences between the studies likely stem from differences in sample sizes. A chi-squared test on the 394 miRNAs tested in both studies indicates that a miRNA identified as an eMiR in one study is more likely to be an eMiR in the other study, *X*^2^(1, *n* = 394) = 9.1, *P* = 2.6 × 10^−3^. Overall, these results demonstrate significant sharing of miRNA genetic regulation between contexts, although important differences in study design and power limit further inference.

### miRNAs contributing to the pathogenesis of brain disorders

GWASs have identified many risk loci for psychiatric and neurologic diseases, but the mechanisms by which these loci confer risk remain unclear. Recently, integrating GWAS signals with gene expression and QTL data has become a powerful strategy for identifying genes linked to complex traits, even when expression measures are unavailable in the larger GWAS participants. In the present study, we leveraged our miRNA expression data and miR-QTL results to uncover genetic variants that confer risk for psychiatric or neurodegenerative diseases through their effects on brain miRNAs.

To do so, we integrated the genome-wide miRNA profiles with results from GWASs of these brain traits using multiple independent and complementary approaches (Fig. 1). First, we performed a miRNA-wide association study (miRWAS) using FUSION^35^, using the miR-QTL results from the reference ROS/MAP dataset to impute genetically regulated miRNAs in the larger GWAS participants and identify miRNAs associated with the brain trait. Next, we determined whether these miRNAs and the brain trait share causal variants through co-localization analysis using coloc^20^. Subsequently, we performed SMR^28^ to verify that the identified miRNAs mediated the association between the GWAS loci and the brain trait, ruling out association due to LD with the HEIDI test^28^ (Fig. 1). In other words, a miRNA was deemed consistent with a causal role in the brain trait if it met the following four criteria: (1) association with the brain trait in the miRWAS at FDR *P* < 0.05; (2) evidence of a shared causal variant indicated by coloc posterior probability of a shared causal variant (PP.H4) > 0.5; (3) evidence for mediating the association between the GWAS locus and brain trait in the SMR analysis (*P* < 0.05); and (4) evidence of a single causal variant based on the HEIDI test (*P* ≥ 0.05). For simplicity, these miRNAs are hereafter referred to as causal miRNAs.

We used data from the latest GWAS summary statistics in five neurodegenerative diseases (Alzheimer’s disease^36^, frontotemporal dementia^37^, amyotrophic lateral sclerosis^38^, Lewy body dementia^39^ and Parkinson’s disease^40^) and 11 psychiatric disorders (major depressive disorder^41^, bipolar disorder^42^, schizophrenia^43^, anxiety^44^, post-traumatic stress disorder (PTSD)^45^, alcoholism^46^, neuroticism^47^, insomnia^48^, attention deficit/hyperactivity disorder^49^, autism^50^ and suicide attempt^51^) (Supplementary Table 13). Among the 470 available miRNAs, 140 had significant heritability (defined as heritability *P* < 0.01) and were included in the miRWAS (Supplementary Table 14).

**Fig. 4:**
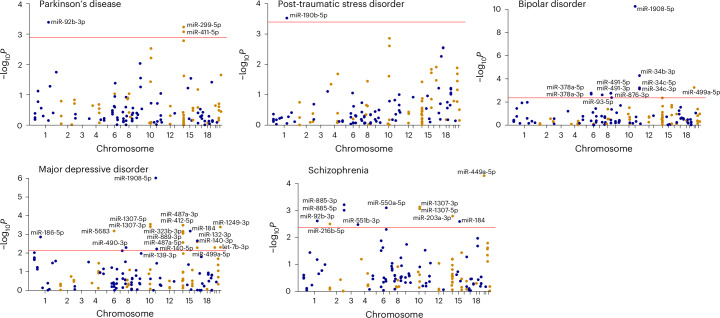
Manhattan plots for the five traits with associated miRNAs in the miRWAS analysis. The *y* axis represents unadjusted FUSION *P* values. Significantly associated miRNAs (at FDR < 5%, indicated by the horizontal red line) are labeled.

We identified 49 unique miRNAs associated with one or more brain traits in the miRWAS (Fig. 4 and Supplementary Tables 15 and 16). Further imposing the remaining three criteria, we found four unique miRNAs consistent with a causal role in Parkinson’s disease (miR-92b-3p), bipolar disorder (miR-1908-5p and miR-499a-5p), major depressive disorder (miR-1908-5p), PTSD (miR-190b-5p) and schizophrenia (miR-499a-5p) (Table 2). Two miRNAs were causal in two brain traits: miR-1908-5p in both bipolar disorder and major depressive disorder and miR-499a-5p in both bipolar disorder and schizophrenia (Table 2).

**Table 2:** Causal miRNAs in psychiatric and neurodegenerative diseases

miRNA	Chr	Median RPM	Trait	miRWAS statistics			COLOC PP4	SMR *P* value	HEIDI *P* value
				Z	*P* value	FDR			
miR-499a-5p	20	325.2	Bipolar disorder	3.5	5.60 × 10^−04^	2.10 × 10^−02^	0.61	3.60 × 10^−02^	0.11
			Schizophrenia	4.1	5.10 × 10^−05^	7.20 × 10^−03^	0.54	2.00 × 10^−03^	0.82
miR-92b-3p	1	1587.6	Parkinson’s disease	−3.5	4.10 × 10^−04^	3.90 × 10^−02^	0.84	1.30 × 10^−03^	0.10
miR-190b-5p	1	1.4	PTSD	−3.6	3.00 × 10^−04^	4.00 × 10^−02^	0.55	3.90 × 10^−03^	0.62
miR-1908-5p	11	2.1	Bipolar disorder	6.6	5.40 × 10^−11^	7.50 × 10^−09^	0.76	3.60 × 10^−10^	0.17
			Major depressive disorder	4.9	9.60 × 10^−07^	1.30 × 10^−04^	0.96	2.30 × 10^−06^	0.91

The miRWAS statistics and COLOC PP4 were estimated using FUSION^35^. The SMR and HEIDI *P* values were estimated using SMR^28^. Chr, chromosome; FDR, false discovery rate corresponding to *P* value; *P*, unadjusted *P* value.

### Examining multi-omics signals in brain disorders

To determine whether the causal roles identified could be attributed to the miRNAs instead of nearby genes, we explored their relationship with a set of causal transcripts and proteins linked to a range of psychiatric and neurodegenerative diseases from a recent study that we conducted^26^. In that study, GWAS summary statistics for brain traits were integrated with brain transcriptomes (*n* = 888) and proteomes (*n* = 722) using transcriptome-wide association study/proteome-wide association study (TWAS/PWAS), Mendelian randomization and co-localization analyses to identify mRNAs and proteins consistent with a causal role in the brain traits^26^. Among the four causal miRNAs that we identified here, three are located within 500 kb of one or more causal transcripts or proteins identified in the previous work.

**Fig. 5:**
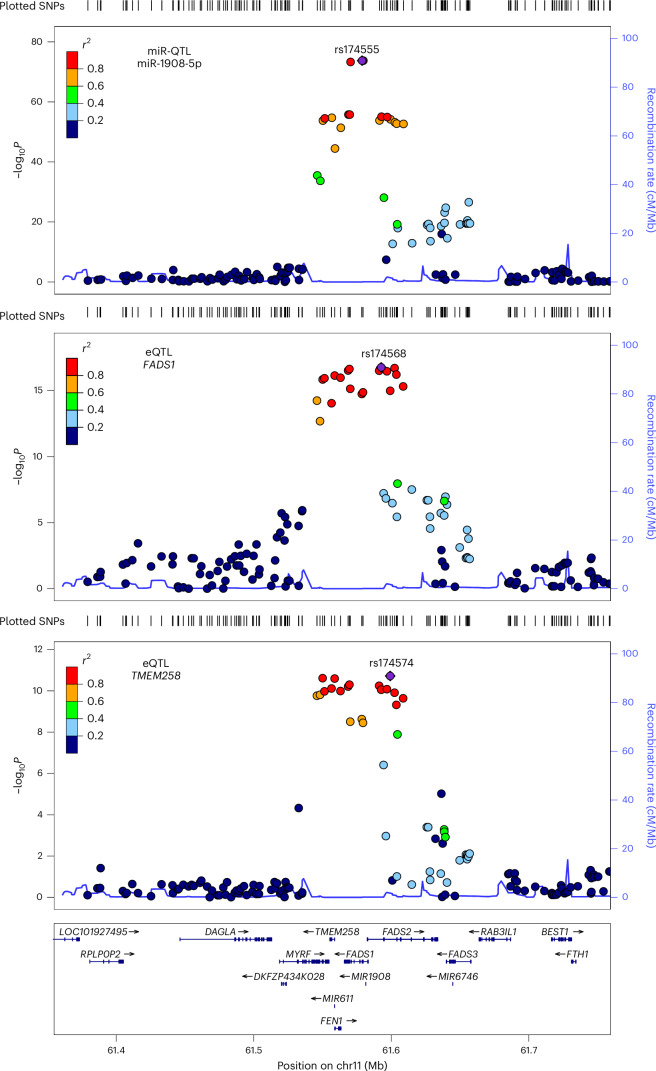
LocusZoom plots for miR-1908-5p miR-QTLs and *FADS1* and *TMEM258* eQTLs. The *y* axes represent unadjusted *P* values from linear regression to test SNP association with miRNA or transcript abundance. In each plot, the SNP with the lowest *P* value is colored purple, and the remaining SNPs are colored according to the extent of LD with that SNP. SMR and HEIDI analysis found that miR-1908-5p miR-QTLs co-localized with *FADS1* eQTLs but not *TMEM258* eQTLs. cM, centimorgan.

**Extended Data Fig. 4:**
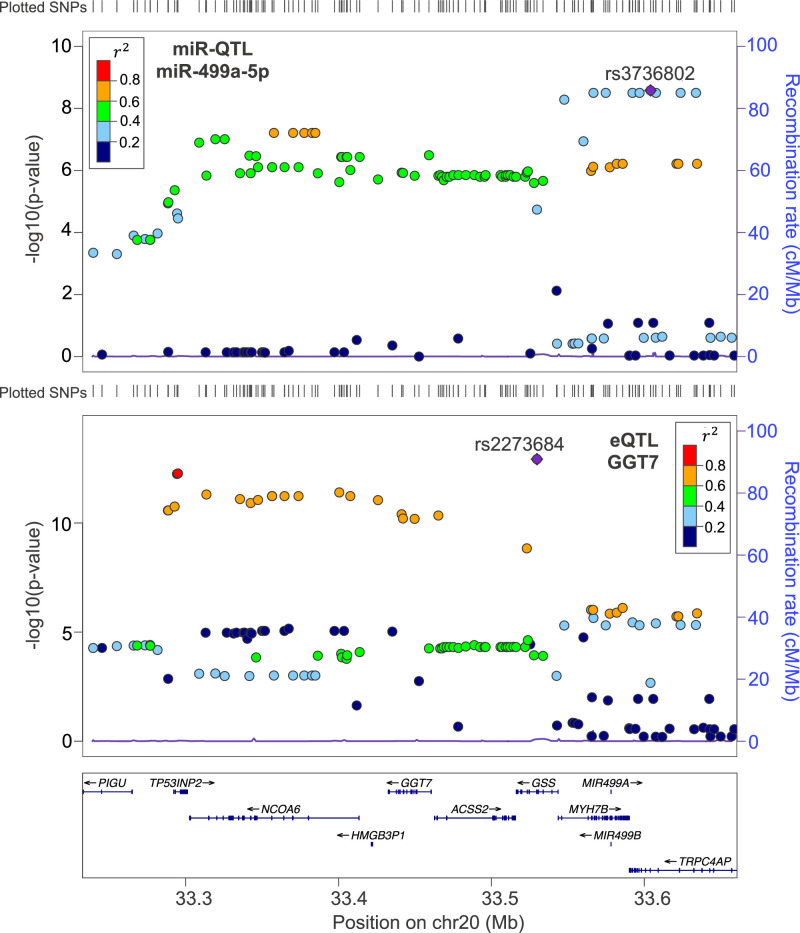
LocusZoom plots for miR-499a-5p miR-QTL and GGT7 eQTL. Y-axes represent unadjusted p-values from linear regression to test SNP association with miRNA or transcript abundance.

**Extended Data Fig. 5:**
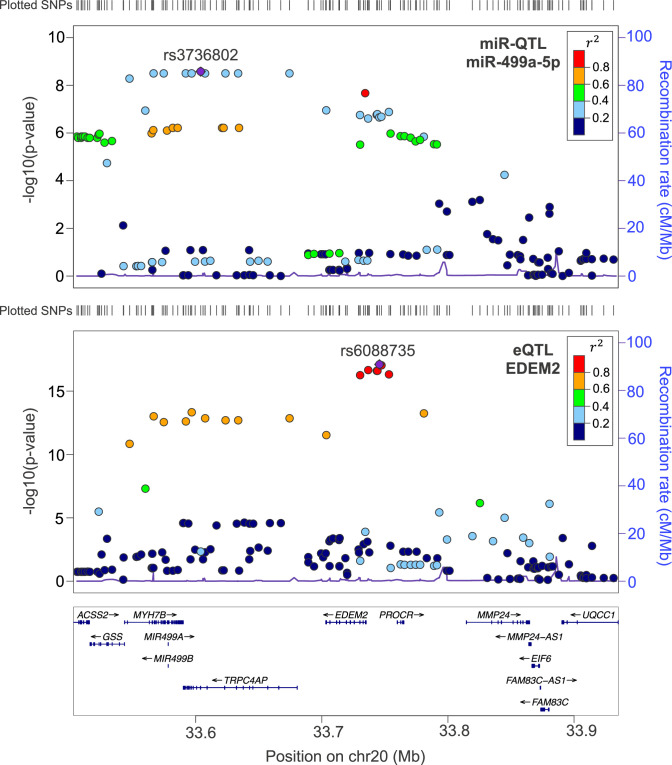
LocusZoom plots for miR-499a-5p miR-QTL and EDEM2 eQTL. Y-axes represent unadjusted p-values from linear regression to test SNP association with miRNA or transcript abundance.

**Extended Data Fig. 6:**
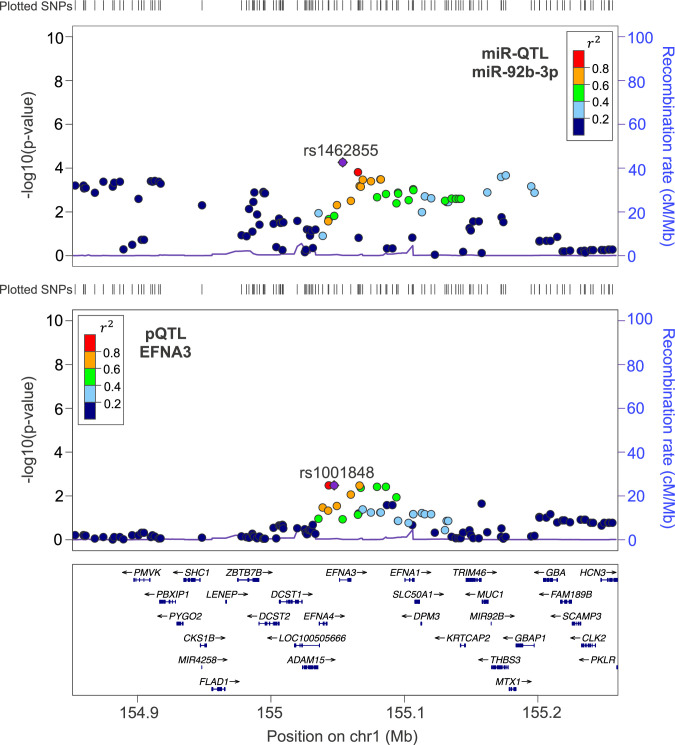
LocusZoom plots for miR-92b-3p miR-QTL and EFNA3 pQTL. Y-axes represent unadjusted p-values from linear regression to test SNP association with miRNA or transcript abundance.

Specifically, miR-1908-5p, causal in both bipolar disorder and major depression, is located within *FADS1* and is close to *TMEM258*, both of which are causal mRNAs for these disorders^26^ (Fig. 5 and Supplementary Table 17). Using SMR for two molecular traits, we found that miR-1908-5p mediates the association between its miR-QTLs and *FADS1* but not with *TMEM258* (Supplementary Table 17). As noted above, the miR-QTLs for miR-1908-5p co-localize with *FADS1* eQTLs, and the miRNA and transcript abundances are negatively correlated. This suggest that miR-1908-5p likely contributes to bipolar disorder and major depression through its effect on *FADS1* mRNA expression but acts independently of *TMEM258*. Similarly, miR-499a-5p is an independent causal mediator from *GGT7* and *EDEM2* in bipolar disorder (Extended Data Figs. 4 and 5 and Supplementary Table 17). Likewise, miR-92b-3p is an independent casual mediator from *EFNA3* in Parkinson’s disease (Extended Data Fig. 6 and Supplementary Table 17). In summary, except for miR-1908-5p, the other three causal miRNAs are causal mediators independent of nearby causal transcripts and proteins, offering biological insights into the pathogenesis of these psychiatric and neurodegenerative diseases.

## Discussion

Using more than 600 small RNA sequencing profiles from dlPFC of older adults, we identified miR-QTLs for 224 miRNAs and integrated those results with human brain proteomes and GWAS summary statistics to investigate how genetically regulated brain miRNAs impact protein expression and neurodegenerative and psychiatric conditions. The adult human brain miR-QTLs that we report here complement genome-wide miR-QTL mapping efforts that were conducted in other human tissues (blood^33,52^, fetal brain^34^, adipose^53^ and liver^54^).

We conducted parallel miR-QTL analyses using reads mapped to two miRNA annotation sets: the popular miRBase database (release 22.1) and the curated MirGeneDB database (version 2.1). MirGeneDB contains fewer than 30% of the annotations in miRBase due to its stricter inclusion criteria. However, applying a minimum abundance filter, we observed substantial overlap, with 75% of miRNA tested in both analyses, as many annotations specific to miRBase were lowly expressed in our brain samples. We opted to use the MirGeneDB set for the subsequent co-localization and integration analyses to enhance the power and interpretability of our findings. Nonetheless, we recognize the potential value of the more comprehensive miRNA set in other contexts and have made all our data available for future studies.

We identified *cis*-miR-QTLs for almost half of the tested miRNAs, similar to 53% of the proteins having pQTLs at FDR < 1% in a recently published study in 716 individuals^26^. Because many miRNAs are located within host genes, a pertinent question is how often these intragenic miRNAs are co-regulated with their host genes. Previous investigations explored this using various methods, such as examining TSS sharing and evaluating co-expression in cell lines^55^. Our co-localization analysis, which used population variation in miRNA expression, host gene expression and genotypes, complements these approaches. We found that most intragenic miRNAs were not genetically co-regulated with their host genes, suggesting that miRNAs have biological roles distinct from their host genes. This also highlights that miR-QTL mapping detects potentially important genetic variants that would not be found in other QTL studies, even for miRNAs located within protein-coding genes.

QTLs for master regulators such as miRNAs can have an outsized effect by influencing the expression of multiple downstream genes. We leveraged our miR-QTL results to identify brain traits and proteins affected by genetically regulated miRNA abundance, identifying four miRNAs consistent with a causal role for complex brain traits. Our findings that miR-1908-5p and miR-499a-5p are causal in bipolar disorder align with a previous candidate gene-based association study^56^. Notably, the miR-92b that we identified to be causal in Parkinson’s disease was shown to target *PTEN*, promote neurite development^57^ and maintain neuroblast self-renewal^58^. Our study complements this existing literature and leverages human brain miRNA expression profiles to provide support for a causal role of these miRNAs. Additionally, we present evidence for the contribution of miR-1908-5p in major depression, miR-190b-5p in PTSD, miR-499a-5p in schizophrenia and miR-92b-3p in Parkinson’s disease. These causal miRNAs present avenues for exploring the molecular mechanisms underlying these neurodegenerative and psychiatric conditions.

We observed 18 miRNA–target gene pairs (involving 16 unique miRNAs) where the miR-QTLs also were pQTLs for the predicted target in the same tissue. Using the SMR and HEIDI tests, we found that six miRNA–target pairs were consistent with pleiotropy or causality. Our subsequent validation experiments confirmed potential interaction for all four pairs tested. Although further investigation is necessary to confirm endogenous repression activity and the observed QTL associations, these findings demonstrate how miR-QTLs can help prioritize putative *trans*-pQTLs and provide additional support for predicted interactions.

We observed significant enrichment of miR-QTLs in promoters active in human brain^31^. To minimize confounding effects between distance to nearest gene and likelihood of a genomic region containing a promoter, we limited our analysis to SNPs within 50 kb of miRNA genes^59^. Our search focused solely on direct overlap with promoters; future analyses could integrate miR-QTLs with chromatin interaction maps to identify those that are distal to promoters but still interact with them^60^.

We unexpectedly found significant depletion of miR-QTLs in microglia enhancers. Although promoter and enhancer detection was conducted using pooled sorted single nuclei^31^, ensuring that the relative cell type proportions did not bias the distributions, our analysis used bulk tissue, where microglia are a minor component. The observed depletion in microglia enhancers is likely an artifact arising from the mutually exclusive nature of enhancers across cell types, low power to detect miR-QTLs specific to microglia cells, and enrichment of miR-QTLs in enhancers of other cell types. Also, the promoter and enhancer annotations were made using data from only 10 individuals and may have missed regulatory regions whose activity varies across individuals^31^.

Comparing our miR-QTLs with those detected in fetal (mid-gestation) neocortex and adult whole blood showed a higher than expected level of eMiR sharing. However, substantial differences among the three studies—including sample size, miRNA quantification method, number of miRNAs analyzed and analytic approach—influence miRNA-QTL detection. Among these factors, variation in sample sizes is likely the most salient, complicating direct comparisons. To help clarify which eMiRs are unique or common across contexts, future studies should include large samples that span different ages and tissue types.

We recognize several limitations of this study. First, the findings would benefit from replication in an independent sample. Although our analysis is bolstered by a relatively large sample size for miRNA profiles, there is currently no independent comparable brain miRNA dataset available for replication. Second, although we characterized miRNA expression in a sizable sample, all the participants were of European ancestry, and expanding studies to include individuals of diverse ancestry is imperative. Third, brain miRNA genetic regulation may differ across stages of adulthood and ought to be tested in future studies that encompass a wider age range. Nevertheless, our observations fill a gap in the available resources for studying the role of miRNAs in the human brain. Furthermore, our analyses focused on a specific brain region (dlPFC), and our observations may not be characteristic of other brain regions. Additionally, some tested miRNAs had modest expression in our dlPFC bulk tissue samples; their cell type-specific expression should be quantified in relevant cell types. For instance, miR-1908-5p was previously observed to be differentially expressed in blood in the acute bipolar state compared with the remission state^61^, suggesting its potential relevance to bipolar disorder despite its relatively low abundance in our study. Finally, by focusing on the manually curated miRNAs in MirGeneDB, we may have overlooked some true miRNAs that did not meet the criteria of that database. The tradeoff for this choice is that we have confidence in the validity of the miRNAs that we did consider, thereby strengthening the signal in our observations and providing a solid foundation to build on in future downstream analyses^11^.

We also highlight several strengths of this study. First, we minimized the impact of cell type variation by estimating surrogate variables (SVs) and including these as covariates in the regression models for miR-QTL mapping. These SVs were designed to capture unknown, unmeasured or hidden confounders, including cell type heterogeneity. Second, we leveraged proteomic data to examine the relationship between the genetic regulation of miRNAs and their downstream targets. Proteomic data may better capture the effect of miRNA post-transcriptional regulation on target genes than transcriptomic data, which is more commonly used. Finally, to our knowledge, this study represents the largest set of genome-wide miRNA sequencing profiles from human brain. The size of our dataset enabled us to conduct well-powered miR-QTL analysis and integrate these results with GWAS data, thereby identifying miRNAs contributing to the pathogenesis of psychiatric and neurodegenerative diseases. The miRNA expression data and catalog of miR-QTL results have been made available for further research. Given the critical regulatory role of miRNAs, these data constitute a valuable resource for future studies aimed at characterizing the complex networks that link population genetic variation to brain-related traits and disease.

## Methods

### Cohorts

The ROS and the MAP are US-based longitudinal community-based cohort studies of cognitive decline and aging^62^. An institutional review board of Rush University Medical Center approved both studies, and we complied with all relevant ethical regulations. All participants provided written informed consent, underwent annual cognitive evaluations, were organ donors and signed an Anatomical Gift Act and repository consent to allow their data and biospecimens to be repurposed. The ROS enrolls priests, monks and nuns from sites across the country, and the MAP enrolls individuals from retirement communities, social service agencies and church groups in the greater Chicago area.

The Arizona Study of Aging and Neurodegenerative Disorders, conducted by the Banner Sun Health Research Institute, primarily recruits cognitively normal individuals from retirement communities in Phoenix, Arizona, and also some individuals with Alzheimer’s disease or Parkinson’s disease^63^. Participants or their legal representatives sign an informed consent form approved by the Banner Sun Health Research Institute institutional review board allowing for brain donation and use of donated biospecimens for approved future research and genetic studies. Participants received no monetary compensation.

### ROS/MAP phenotypes

Clinical diagnosis at death was based on the opinion of a neurologist with expertise in dementia who had access to select clinical but not postmortem data. Sex (male or female) was based on self-report. Postmortem interval (PMI) is the time interval from time of death to autopsy. Self-identified race was defined based on the participant’s response to the question ‘What is your race?’ with possible answers including White; Black or African American; American Indian or Alaska Native; Native Hawaiian or Other Pacific Islander; Asian; or Other.

### miRNA sequencing

Frozen postmortem dlPFC tissue samples from 743 brains were obtained from Rush University. RNA extraction from the tissue samples was performed with TRIzol (Invitrogen) following the manufacturer’s protocol. Approximately 80 mg (range, 50–100 mg) of frozen brain tissues was used to extract RNA. RNA quality was measured by two methods following the manufacturer’s protocol: (1) NanoDrop spectrophotometer (Thermo Fisher Scientific) and (2) Bioanalyzer RNA 6000 Nano (Agilent). NanoDrop spectrophotometer measures concentration and A_260_ / A_280_ ratio, which determines the purity of RNA. The average concentration of extracted RNA samples was approximately 900 ng µl^−1^ resuspended in 50 µl of DEPC-treated water, and the average A_260_ / A_280_ ratio was 1.93. Bioanalyzer RNA 6000 Nano was used to determine an RNA integrity number (RIN) to assess RNA quality. The average RIN score for all 743 RNA samples was 5.5; 672 samples with RIN > 2.1 were selected for miRNA library preparation. Based on RIN, PMI, sex, cognitive diagnosis, amyloid, tangles, depression, education and sense of life purpose, samples were randomized into batches of eight for miRNA library preparation.

miRNA library preparation was conducted using NEBNext Muliplex Small RNA Library Prep Kit for Illumina (Index Primers 1–48) (New England Biolabs (NEB)) following the manufacturer’s protocol. One microgram of total RNA was used as input for the miRNA library preparation in batches of 48. Libraries were checked for the presence of a 21-nt insert using a Bioanalyzer DNA 1000 before size selection with a Pippin Prep (Sage Science). The molarity of each miRNA library was measured by a Qubit fluorometer (Thermo Fisher Scientific), and libraries were diluted and pooled. miRNA sequencing was performed using an Illumina HiSeq 3000 at the Emory Yerkes Genomics Core.

### miRNA quantification

Adaptor sequences were trimmed using Trimmomatic^64^ (version 0.36), and miRNA counts were generated using mirDeep2 (ref. ^65^). Specifically, we first used ‘mapper.pl‘ from mirDeep2 to generate collapsed FASTA files and then removed reads with length less than 20 nt or more than 25 nt using a custom script. Then, ‘quantifier.pl‘ from mirDeep2 was used to map the reads to known human precursor and mature miRNA sequences obtained from MiRBase^12^ (release 22.1). Reads were allowed to map up to 2 nt upstream of the mature sequence and up to 5 nt downstream of the mature sequence and with up to one mismatch. These are the default parameters for mirDeep2 and are reasonable because they allow for untemplated nucleotide addition and imprecise processing, which are common especially at the 3′ end of mature miRNAs.

Because miRBase was reported to contain a substantial number of potentially false-positive miRNAs^66^, we performed a second mapping run after filtering the reference precursor and mature miRNA FASTA files to only the 504 precursors and 857 mature miRNAs present in MirGeneDB^13^ (version 2.1), a database of miRNAs that meet a set of criteria designed to distinguish true miRNA sequences from false annotations and other types of small RNAs^14^.

### GWAS summary statistics

We used association summary statistics from GWASs of five neurodegenerative diseases (Alzheimer’s disease^36^, frontotemporal dementia^37^, amyotrophic lateral sclerosis^38^, Lewy body dementia^39^ and Parkinson’s disease^40^) and 11 psychiatric traits (major depressive disorder^41^, bipolar disorder^42^, schizophrenia^43^, anxiety^44^, PTSD^45^, alcoholism^46^, neuroticism^47^, insomnia^48^, attention deficit/hyperactivity disorder^49^, autism^50^ and suicide attempt^51^). We focused on GWAS summary statistics results from samples of individuals with European ancestry (Supplementary Table 13).

### Transcriptomic and proteomic data

Transcriptomic and proteomic profiles from postmortem dlPFC samples were available from a published study^21^. Transcriptomic data included 589 ROS/MAP participants (376 female and 213 male, mean age 89 years (range, 67–108 years)), sequenced using Illumina HiSeq and aligned to GRCh38. Low-abundance transcripts (<1 RPM for ≥50% of samples) were filtered, and normalized log_2_-transformed counts were adjusted for batch effects, RIN, PMI, age, and clinical diagnosis by regressing out these variables.

Proteomic data included 716 ROS/MAP and Banner participants (453 female and 263 male, mean age 89 years (range, 66–108 years)) from previous studies (ROS/MAP proteomics^67^ and Banner proteomics^5^). Each dataset underwent quality control and normalization independently. Specifically, batch-specific controls were used to remove proteins outside the 95% CI, and proteins with missing data for over half the samples were excluded. Outlier samples (>4 s.d. from the mean of the first two principal components (PCs), calculated iteratively until no outliers remained) were removed. Residuals were computed after regressing out mass spectrometry (MS) mode, batch, sex, age, PMI, and clinical diagnosis of cognitive status and then z-scaled and merged.

### Luciferase experiments

To validate the predicted miRNA/mRNA target pair interactions, we transfected human HEK293T cells with pairs of a miRNA mimic and a 3′ UTR reporter construct with firefly luciferase as a reporter as follows: miR-1307-5p with 3′ UTR of PPFIA3; miR-1307-3p with 3′ UTR of PPFIA3; miR-185-3p with 3′ UTR of TANGO2; and miR-1307-5p with 3′ UTR of LYPLA2. Catalog numbers for materials used in transfection and luciferase reporter assays are available in Supplementary Table 18. Transfection was performed using human HEK293T cells, maintained in DMEM supplemented with 10% FBS and 1% antibiotics solution (penicillin–streptomycin) at 37 °C and 5% CO_2_. One day before transfection, cells were plated on two 96-well plates (50,000 cells per well). The cells were transfected using Lipofectamine 2000 (Thermo Fisher Scientific) with the miRNA mimic and a 3′ UTR reporter construct pair as indicated above and incubated for 48 h at 37 °C and 5% CO_2_. Each miRNA mimic/3′ UTR reporter construct pair was transfected in quadruplicate on each plate. Luciferase assay was subsequently carried out with the ONE-Glo EX Luciferase Assay (Promega). Transfected cells were treated in accordance with the manufacturer’s protocols, and luminescence was assayed on a Synergy HTX multi-mode microplate reader (BioTek).

### Statistics and reproducibility

All statistical analyses were done using R version 3.6.0 unless otherwise noted. All genome coordinates are with respect to human reference GRCh37. To validate putative miRNA–target interactions, for each putative interacting pair we compared luminescence between experimental conditions (that is, miRNA + target 3′ UTR plasmid versus vehicle + target 3′ UTR plasmid) using linear regression, including a term to adjust for plate effects. Each condition was represented by eight independent replicates. Significant difference in luciferase expression was defined as unadjusted *P* < 0.05. The other statistical analyses are described in detail in the following sections.

### miRNA and sample filtering

We filtered very lowly expressed miRNAs, which we defined as those with <1 RPM for >50% of samples. We chose a liberal threshold for this filter to allow for the fact that miRNAs can have low observed RPM in our heterogeneous bulk tissue samples but higher expression in specific cell types. For miRNAs mapped to multiple precursors, we kept the entry with the highest total count across samples.

miRNA coordinates were obtained from miRBase version 22 and then converted from GRCh38 to GRCh37 using LiftOver to match the build used for the genotype data and GWAS summary statistics. miRNAs included in the analysis met the abundance threshold described above, had gene coordinate information in the coordinate file, had at least one precursor gene located on an autosome and had at least one common (minor allele frequency (MAF) > 5%) SNP within the test window, which included the precursor sequence(s) plus 500kb symmetric padding around each precursor sequence. For miRNAs with multiple precursors that were fewer than 500 kb apart, SNPs that fell in the padding region for multiple precursors were tested only once for the miRNA.

Sample filtering was performed on several criteria. First, we iteratively removed samples that were more than 5 s.d. from the mean within the respective batch for total read count, trimmed read rate and mapped read rate. We then filtered 11 samples to focus analysis on individuals of European ancestry based on genetic data. The analysis was limited to these participants because of small sample sizes for participants from other genetic ancestries. The raw counts were then normalized for library size and transformed to log_2_ counts using edgeR. PCs were estimated, and samples that were more than 4 s.d. from the mean of either of the first two PCs were removed. Sample characteristics for the 604 participants who were included in the analysis are summarized in Supplementary Table 1.

### miR-QTL mapping

Genotype data used in this analysis were generated in a previous study^68^. DNA was extracted from blood or brain, and genotyping was performed using whole-genome sequencing (WGS) or genome-wide genotyping (Affymetrix GeneChip 6.0 or Illumina OmniQuad Express platforms). We performed initial filtering on the WGS, Affymetrix chip and Illumina chip datasets separately. Specifically, samples were excluded if they had genotype missingness more than 5% or were from participants who self-identified their race as other than White, and variants were excluded if they had evidence of deviation from Hardy–Weinberg equilibrium (*P* < 1 × 10^−8^), had genotype missingness more than 5%, had MAF < 1%, or were not a SNP. Sample outliers with respect to population structure were removed using EIGENSTRAT^69^. For samples with array-based genotypes, imputation was performed to 1000 Genomes Project phase 3 (ref. ^70^) using the Michigan Imputation Server^71^, and SNPs with imputation *r*^2^ > 0.3 were retained. Second-degree and closer relatives were removed using KING^72^. Genotypes for the 604 participants with miRNA data that passed quality control were then extracted from the WGS and imputed chip datasets and merged. Some participants were genotyped using both WGS and chip; in these cases, the WGS genotypes were kept. Before merging, variants with MAF < 5% in each dataset were removed. We then used bcftools to identify sites common to both datasets and with identical reference and alternate alleles and merged the sample data for these sites. The MAF, Hardy–Weinberg equilibrium and genotype missingness checks were repeated after merging. PC analysis confirmed that all samples clustered with the 1000 Genomes Project CEU population.

To identify miR-QTLs, the effect of SNP allele dosage (assuming an additive effect) on miRNA abundance was estimated for each SNP–miRNA pair in turn using the ‘glm’ function from PLINK2 (ref. ^73^). The following variables were included as covariates: miRNA batch, PMI, RIN, age at death, cognitive diagnosis at death (normal versus impaired), study (ROS versus MAP), sex, genotype platform, 10 genetic PCs and 15 miRNA SVs. SVs were estimated from the normalized counts using the R package ‘sva’. The number of SVs to estimate was set at 15 based on the results of the num.sv function, and SVs were estimated with a randomly generated binary variable as the variable of interest and miRNA batch, PMI, RIN, age at death, cognitive diagnosis at death, study (ROS versus MAP) and sex as adjustment variables. The genetic PCs were calculated using PLINK2. We observed that the genetic PCs were strongly correlated with genotype platform. We set the maximum variance inflation factor to 500 to allow the tests to run despite multicollinearity between these variables. *q* values were calculated using the R package ‘qvalue’ using *P* values from all tests. We defined miR-QTLs as SNP–miRNA pairs with *q* < 0.01.

Because we performed the miR-QTL mapping using dense genotyping, many miR-QTLs are in high LD and represent essentially the same genetic signal. We extracted a reduced set of miR-QTLs for each miRNA using the ‘clump’ function from PLINK1.9. This function applies a greedy algorithm to iteratively prune miR-QTLs to arrive at a set of ‘index’ miR-QTLs that have pairwise *r*^2^ < 0.5 or are separated by at least 250 kb.

### miR-QTL characterization

The percent of miRNA variance explained by individual miR-QTLs was estimated using the R package ‘variancePartition’. The outcome in the model was the residuals after regressing the effects of miRNA batch, PMI, RIN, age at death, cognitive diagnosis, study, sex and miRNA SVs from the normalized expression profiles, and the predictors were SNP and 10 genetic PCs.

For each miRNA with more than one index SNP, we used conditional analysis to identify miR-QTLs with independent signal. First, we ranked the miR-QTLs for each miRNA by increasing *P* value. The independent variants list was initiated with the miR-QTL with the lowest *P* value. For each remaining miR-QTL, an association test was run using the same model as the initial analysis except with the independent variant(s) also included as predictors. If more than one miR-QTL remained significant, the top-ranked miR-QTL was added to the independent variants list, and another round of testing was performed with the remaining miR-QTLs. If only one miR-QTL was significant, it was added to the independent variants list, and the procedure was complete.

We defined miRNAs as intragenic if they overlapped and were on the same strand as a protein-coding gene, long non-coding RNA or short non-coding RNA other than the miRNA gene of interest, and as intergenic otherwise. Gene coordinates and strand information were obtained from Ensembl. miRNA clusters (miRNA genes with inter-miRNA distance <10 kb) were copied from the miRBase website. Any precursor that was absent from the miRBase clusters was assigned to its own cluster.

### Co-localization with host gene eQTLs and pQTLs

Correlation between miRNA and transcript abundance was estimated using Spearman correlation after regressing out batch, PMI, RIN, sex, age at death, study, clinical diagnosis of Alzheimerʼs disease and SVs from normalized miRNA data and regressing out batch, PMI, RIN, sex, age at death and clinical diagnosis and SVs from normalized transcript data.

For intragenic miRNAs, we characterized co-localization between miR-QTLs and host gene QTLs using the R package ‘coloc’. *Cis*-eQTL and *cis*-pQTL summary statistics were estimated using the transcriptomic, proteomic and corresponding genotype data (ROS/MAP genotypes^68^ and Banner genotypes^5^) with linear regression adjusted for transcriptomic/proteomic SVs. We applied the SuSiE wrapper to identify credible sets of causal variants for the miR-QTL and host gene QTL datasets separately. Diagnostic plots were inspected to verify allele matching between the summary statistics and genotype data used to estimate LD. Then, following the recommendation in ref. ^74^, we tested for co-localization using a hybrid approach, allowing for multiple causal variants using the function coloc.susie if credible sets were identified for both datasets and assuming a single casual variant using the function coloc.abf otherwise. Co-localization was defined as posterior probability of a shared causal variant (PP.H4) > 0.5.

### Association testing and SMR for predicted target genes

Predicted targets for each eMiR were gathered using TargetScan 7.2 and RNA22 version 2. For TargetScan, we downloaded context++ scores and selected targets with cumulative weighted context++ score < −0.2, as more negative scores indicate stronger targeting efficacy. For conserved miRNAs, we kept only targets with at least one conserved site. For RNA22, we used genes from our proteomics dataset and obtained the MANE transcript for each from GENCODE. We then used the RNA22 batch submission program with the following parameters: sensitivity of 63%/specificity of 61%; seed size of 7 with 0 unpaired bases in seed; minimum 12 paired-up bases in heteroduplex; maximum folding energy of −12 kcal mol^−1^ for heteroduplex; and no limit on number of G:U wobbles in the seed region. We kept predictions with *P* < 0.05. We took the union of targets from the TargetScan and RNA22 predictions, with a median of 586 predicted target genes per miRNA (IQR 291–1,053).

pQTL analysis was conducted using the combined proteomics dataset, which included SNP genotypes and dlPFC proteomic profiles from 716 ROS/MAP and Banner study participants. Of the 716 participants, 565 were from the ROS/MAP cohort, and 511 of those overlapped the 604 participants included in the miR-QTL analysis. Sample size varied across different proteins from 76 to 716 participants due to differences in missingness rate across proteins. We used the ‘glm’ function from PLINK2 to test the effect of miR-QTL allele dosage on protein abundance of the predicted targets. Proteomic profiles were represented by residuals after regressing out effects of batch, PMI, age at death and clinical diagnosis. The pQTL models included sex, protein SVs and 10 genetic PCs as covariates. We performed multiple testing correction for the approximately 17,400,000 SNP–protein tests and defined pQTLs at FDR < 5%.

For miR-QTLs that were also pQTLs for predicted target proteins, we used SMR^28^ to test whether the genetically regulated miRNA abundance mediates the effect of the miR-QTL on protein abundance. miR-QTL and pQTL summary statistics were used as the exposure and outcome, respectively. SMR significance was defined as Bonferroni-corrected *P* < 0.05 using the number of SMR tests conducted. SMR association can arise from causality or pleiotropy of a single SNP that affects both the exposure and outcome or from linkage of SNPs with distinct effects on the exposure and outcome. We used HEIDI^28^ to identify signals arising from causality or pleiotropy (HEIDI *P* ≥ 0.05) rather than linkage (HEIDI *P* < 0.05).

### Enrichment in gene promoters and enhancers

To test for enrichment of miR-QTLs in brain promoters and enhancers, the Cochran–Mantel–Haenszel test (function mantelhaen.test from the R base package ‘stats’) was applied using three MAF strata: (0–0.1), [0.1–0.2), [0.2–0.5]. A 2 × 2 table was constructed within each MAF strata (that is, ‘Is the SNP a miR-QTL?’ versus ‘Does the SNP overlap a promoter?’). For enhancers, SNPs in the full test window (500 kb symmetric padding around the miRNA gene) were considered. For promoters, SNPs within 50 kb of miRNA precursors that were tested in our study were considered. This window was chosen based on the expected proximity of promoters to the gene and given that miRNAs may be found in clusters or within host genes^75^. Significant enrichment was declared at unadjusted *P* < 0.05.

### Comparison with other tissues

The SNPs in the fetal neocortex miR-QTL study^34^ were reported by genomic position with respect to human reference GRCh38. To match them with our results, we applied LiftOver to convert SNP positions to GRCh37 coordinates. SNPs in the blood miR-QTL study were matched based on rsID.

For each miRNA with a miR-QTL in the discovery set (that is, the fetal neocortex set or the blood set), we selected the miR-QTL with the lowest *P* value and looked up its *P* value, if available, in the replication set (our adult dlPFC set). We used these *P* values to calculate the ${\pi }_{1}$ statistic, representing the fraction of true positives, as 1-*π*_0_, where ${\pi }_{0}$ is the fraction of true negatives estimated using function ‘qvalue’ from the R package qvalue.

### Integration with GWASs

We integrated human brain genome-wide miRNA profiles with GWAS results for brain traits using multiple independent but complementary approaches. First, we performed a miRWAS using FUSION^35^, estimating miRNA weights from the reference ROS/MAP dataset including both genetic and miRNA expression data. These miRNA weights were then integrated with GWAS summary statistics of neurodegenerative and psychiatric traits. For the weight calculation, we restricted the genome-wide genotypes to an LD reference panel of 1,190,321 SNPs provided by FUSION. SNP-based heritability for each miRNA was estimated, and, for miRNAs with heritability *P* < 0.01, FUSION used multiple predictive models (top1, blup, lasso, enet and bslmm) to compute the effect of SNPs on miRNA abundance, selecting the most predictive model. SNPs within a 500-kb window around each miRNA were used to calculate the linear sum of z-score × weight to perform miRWAS.

Next, we used co-localization analysis with coloc^20^ to assess whether identified miRNAs and brain traits share causal variants. FUSION provides an interface to the coloc software to compute an approximate co-localization statistic based on the marginal FUSION weights^35^. We used coloc to estimate the posterior probability that the miRNA and traits share a causal variant (PP.H4).

We then performed SMR^28^ to verify that the identified miRNAs mediated the association between GWAS loci and brain traits, using the HEIDI test^28^ to rule out association due to LD.

In summary, a miRNA was considered consistent with a causal role in a brain trait if it met these four criteria: (1) association with the brain trait in the miRWAS at FDR *P* < 0.05; (2) evidence of a shared causal variant indicated by coloc posterior probability of a shared causal variant (PP.H4) > 0.5; (3) evidence for mediating the association between the GWAS locus and brain trait in the SMR analysis (*P* < 0.05); and (4) evidence of a single causal variant based on the HEIDI test (*P* ≥ 0.05).

### Mediation analysis of overlapping multi-omics signals

Among the four identified causal miRNAs in brain illnesses, two were regulated by miR-QTLs in proximity (±500 kb window) with eQTLs of causal mRNAs for the same brain illness, and one was regulated by miR-QTLs in proximity with pQTLs for a causal protein for the same brain trait (Supplementary Table 17). These causal mRNAs and proteins are from a recently published work^26^. Because of their proximity, we sought to determine whether the causal miRNAs mediated the association between miR-QTLs and causal mRNAs or protein, respectively, for a particular brain trait using SMR for two molecular traits^76^. We used miR-QTL summary data as the exposure and eQTL or pQTL summary data as the outcome. Mediation was defined as SMR *P* < 0.05 and HEIDI *P* ≥ 0.05.

### Reporting summary

Further information on research design is available in the Nature Portfolio Reporting Summary linked to this article.

## Supplementary information

Reporting Summary

Supplementary Tables 1–18

## Data Availability

The new and existing datasets used in this study are deposited in Synapse (https://www.synapse.org/). All datasets require creating a Synapse user account. miRNA data and miR-QTL summary statistics presented in this paper can be found with Synapse ID syn51247298. Transcriptomic data and eQTL and pQTL summary statistics used for host gene co-localization can be found with Synapse ID syn51150434. All data not restricted to protect patient privacy are also available from the corresponding authors upon reasonable request. Genotype and proteomic data are under controlled access to protect patient privacy and can be obtained by submitting a Synapse data access request, including an intended use statement and a data use certificate. Application review time is 1–2 weeks. The Synapse IDs for each dataset are ROS/MAP genotype data: syn17008936; Banner genotype data: syn24875702; ROS/MAP proteomics: syn21238468; and Banner proteomics: syn9884314. ROS/MAP resources can also be requested at https://www.radc.rush.edu. Web resources: miRBase miRNA clusters: https://www.mirbase.org/cgi-bin/mirna_summary.pl?org=hsa&cluster=10000, accessed 22 August 2022 TargetScan release 7.2: http://www.targetscan.org/cgi-bin/targetscan/data_download.vert72.cgi PsychEncode reference enhancer list: http://resource.psychencode.org

## Code availability

The analyses were conducted using publicly available software and R packages. Conversion between genome builds was done using LiftOver (downloaded January 2021, https://genome-store.ucsc.edu/). Adapter trimming of miRNA reads was done using Trimmomatic version 0.36 (http://www.usadellab.org/cms/?page=trimmomatic). miRNA quantification was done using mirDeep2 version 0.1.2 (https://github.com/rajewsky-lab/mirdeep2/). Genotype filtering and VCF manipulation were done using vcftools version 0.1.13 (https://vcftools.github.io), bcftools version 1.9 (https://github.com/samtools/bcftools) and PLINK version 2.0a (https://www.cog-genomics.org/plink/2.0/). Identification of population outliers was done using EIGENSTRAT version 6.1.4 (https://github.com/DReichLab/EIG). Linear regression to identify miR-QTLs was done using PLINK version 2.0a. Clumping of miR-QTL signals was done using PLINK version 1.90b53 (https://www.cog-genomics.org/plink/1.9/). Genomic interval overlaps were characterized using bedtools version 2.27.0 (https://github.com/arq5x/bedtools2). Target prediction was done using RNA22 version2 (https://cm.jefferson.edu/rna22/Interactive/). Summary data-based Mendelian randomization was done using SMR version 1.03 for miR-QTLs with target protein expression and version 1.02 for miR-QTLs with GWAS traits (https://yanglab.westlake.edu.cn/software/smr/). miRWAS and co-localization analysis of miR-QTLs and GWAS traits was done using FUSION (including the FUSION interface to pass marginal FUSION weights to coloc) (http://gusevlab.org/projects/fusion/). Other analyses were done using R (version 3.6.0, unless otherwise noted) using base functions or the following R packages: data.table version 1.14.2 for data manipulation; edgeR version 3.28.1 to normalize miRNA data; variancePartition version 1.16.0 to estimate percent variance explained; sva version 3.34.0 for surrogate variable analysis; coloc version 5.2.1 (using R version 4.1.1) for co-localization analysis of miR-QTLs and host gene eQTLs; and qvalue version 2.15.0 to calculate *q* values and replication statistics. Code is available upon reasonable request.
