# Supplementary material for: Mapping the microRNA landscape in the older adult brain and its genetic contribution to neuropsychiatric conditions
Source: Nat Aging. 2024 Dec 6;5(2):306–19. doi: 10.1038/s43587-024-00778-x (PMC11839474; doi:10.1038/s43587-024-00778-x)
Supplement: Supplementary file 1 — Reporting Summary [file 43587_2024_778_MOESM1_ESM.pdf]

nature portfolio

Corresponding author(s): Aliza P. Wingo
Thomas S. Wingo

Last updated by author(s): 2024/10/25

Reporting Summary

Nature Portfolio wishes to improve the reproducibility of the work that we publish. This form provides structure for consistency and transparency in reporting. For further information on Nature Portfolio policies, see our Editorial Policies and the Editorial Policy Checklist.

Statistics

For all statistical analyses, confirm that the following items are present in the figure legend, table legend, main text, or Methods section.

n/a	Confirmed	
☐	☒	The exact sample size (*n*) for each experimental group/condition, given as a discrete number and unit of measurement
☐	☒	A statement on whether measurements were taken from distinct samples or whether the same sample was measured repeatedly
☐	☒	The statistical test(s) used AND whether they are one- or two-sided<br>*Only common tests should be described solely by name; describe more complex techniques in the Methods section.*
☐	☒	A description of all covariates tested
☐	☒	A description of any assumptions or corrections, such as tests of normality and adjustment for multiple comparisons
☐	☒	A full description of the statistical parameters including central tendency (e.g. means) or other basic estimates (e.g. regression coefficient) AND variation (e.g. standard deviation) or associated estimates of uncertainty (e.g. confidence intervals)
☐	☒	For null hypothesis testing, the test statistic (e.g. *F*, *t*, *r*) with confidence intervals, effect sizes, degrees of freedom and *P* value noted<br>*Give P values as exact values whenever suitable.*
☒	☐	For Bayesian analysis, information on the choice of priors and Markov chain Monte Carlo settings
☐	☒	For hierarchical and complex designs, identification of the appropriate level for tests and full reporting of outcomes
☐	☒	Estimates of effect sizes (e.g. Cohen's *d*, Pearson's *r*), indicating how they were calculated

Our web collection on statistics for biologists contains articles on many of the points above.

Software and code

Policy information about availability of computer code

Data collection: MiRNA sequencing was performed using an Illumina HiSeq 3000.

Data analysis: Analysis was conducted using publicly available software and R packages as noted in the 'Code Availability' section. Conversion between genome builds was done using LiftOver (https://genome-store.ucsc.edu/). Adapter trimming of miRNA reads was done using Trimmomatic v0.36 (http://www.usadellab.org/cms/?page=trimmomatic). miRNA quantification was done using mirDeep2 v0.1.2 (https://github.com/rajewsky-lab/mirdeep2/). Genotype filtering and VCF manipulation was done using vcftools v0.1.13 (https://vcftools.github.io), bcftools v1.9 (https://github.com/samtools/bcftools), and PLINK v2.0a (https://www.cog-genomics.org/plink/2.0/). Identification of population outliers was done using EIGENSTRAT (https://github.com/DReichLab/EIG). Linear regression to identify miR-QTLs was done using PLINK v2.0a. Clumping of miR-QTL signals was done using PLINK v1.90b53 (https://www.cog-genomics.org/plink/1.9/). Genomic interval overlaps were characterized using bedtools v2.27.0 (https://github.com/arq5x/bedtools2). Summary data-based Mendelian randomization was done using SMR v1.03 for miR-QTLs with target protein expression and v1.02 for miR-QTLs with GWAS traits (https://yanglab.westlake.edu.cn/software/smr/). miRWAS and colocalization analysis of miR-QTLs and GWAS traits was done using FUSION (including the FUSION interface to pass marginal FUSION weights to coloc) (http://gusevlab.org/projects/fusion/). Other analyses were done using R (version 3.6.0 unless otherwise noted) using base functions or the following R packages: data.table v1.14.2 for data manipulation; edgeR v.3.28.1 to normalize miRNA data; variancePartition v1.16.0 to estimate percent variance explained; sva v.3.34.0 for surrogate variable analysis; coloc v5.2.1 (using R version 4.1.1) for colocalization analysis of miR-QTLs and host gene eQTLs; qvalue v2.15.0 to calculate qvalues and replication statistics.

For manuscripts utilizing custom algorithms or software that are central to the research but not yet described in published literature, software must be made available to editors and reviewers. We strongly encourage code deposition in a community repository (e.g. GitHub). See the Nature Portfolio guidelines for submitting code & software for further information.

Data

Policy information about availability of data

All manuscripts must include a data availability statement. This statement should provide the following information, where applicable:

- Accession codes, unique identifiers, or web links for publicly available datasets
- A description of any restrictions on data availability
- For clinical datasets or third party data, please ensure that the statement adheres to our policy

miRNA data and miR-QTL summary statistics will be available upon publication on synapse.org at syn51247298. ROS/MAP resources are under controlled access to protect patient privacy and can be requested at https://www.radc.rush.edu.

Research involving human participants, their data, or biological material

Policy information about studies with human participants or human data. See also policy information about sex, gender (identity/presentation), and sexual orientation and race, ethnicity and racism.

Reporting on sex and gender	Sex was based on self-report and confirmed using genotype data. Particularly, biological sex was determined based on the heterozygosity rate across genetic variants located on the X chromosome in each donor. As part of genotyping quality control, we checked the agreement between self-reported gender and genetic sex and they agreed for all subjects included in the analyses.
Reporting on race, ethnicity, or other socially relevant groupings	All participants included in the study self-identified as White in response to the question 'What is your race?' With possible answers White; Black or African American; American Indian or Alaska Native; Native Hawaiian or Other Pacific Islander; Asian; or Other. All included participants were of European ancestry based on genotype data (using principal components to compare participant genotypes with genotypes from the Thousand Genomes CEU population). The analysis was limited to these participants because of small samples sizes for participants from other genetic ancestries.
Population characteristics	The ROS participants were priests, monks, and nuns from sites across the United States, and MAP participants were recruited from retirement communities, social service agencies, and church groups in the greater Chicago area. All participants were over 70 years old at the time of death, and the median age of death was 90 years. About 70% (432 of 604) of the participants were female.
Recruitment	All research participants and post-mortem brain donors signed informed consent.
Ethics oversight	ROS and MAP received approval from an Institutional Review Board of Rush University Medical Center.

Note that full information on the approval of the study protocol must also be provided in the manuscript.

Field-specific reporting

Please select the one below that is the best fit for your research. If you are not sure, read the appropriate sections before making your selection.

☒ Life sciences ☐ Behavioural & social sciences ☐ Ecological, evolutionary & environmental sciences

For a reference copy of the document with all sections, see nature.com/documents/nr-reporting-summary-flat.pdf

Life sciences study design

All studies must disclose on these points even when the disclosure is negative.

Sample size	No sample size calculations were performed. All available samples with brain proteomic, genetic, and phenotypic data that passed quality control were used for the analysis.
Data exclusions	Outlier samples were removed in the quality control step of miRNA data. This was done through an iterative process of detecting outliers by principal component analysis of the proteomic data and excluding all individuals who were greater than 4 standard deviations from the mean of the first two principal components. Data were also filtered to participants who self-reported their race as White and were of European ancestry based on genotype principal component analysis. Then we included individuals with both miRNA and genome-wide genotyping data for the analyses.
Replication	Replication of miR-QTLs was not performed due to lack of availability of a comparable adult brain miRNA dataset. Interaction of miRNAs and predicted targets that were linked in our SMR analysis was experimentally validated via luciferase assays in HEK 293T cells. Interaction was supported for all four tested miRNA-target pairs.
Randomization	Samples were randomized into batches of 8 for miRNA library preparation and sequencing based on RIN, PMI, sex, cognitive diagnosis, amyloid, tangles, depression, education, and sense of life purpose.
Blinding	The individuals preparing samples for miRNA sequencing were blinded to phenotypic information.

Behavioural & social sciences study design

All studies must disclose on these points even when the disclosure is negative.

Study description	*Briefly describe the study type including whether data are quantitative, qualitative, or mixed-methods (e.g. qualitative cross-sectional, quantitative experimental, mixed-methods case study).*
Research sample	*State the research sample (e.g. Harvard university undergraduates, villagers in rural India) and provide relevant demographic information (e.g. age, sex) and indicate whether the sample is representative. Provide a rationale for the study sample chosen. For studies involving existing datasets, please describe the dataset and source.*
Sampling strategy	*Describe the sampling procedure (e.g. random, snowball, stratified, convenience). Describe the statistical methods that were used to predetermine sample size OR if no sample-size calculation was performed, describe how sample sizes were chosen and provide a rationale for why these sample sizes are sufficient. For qualitative data, please indicate whether data saturation was considered, and what criteria were used to decide that no further sampling was needed.*
Data collection	*Provide details about the data collection procedure, including the instruments or devices used to record the data (e.g. pen and paper, computer, eye tracker, video or audio equipment) whether anyone was present besides the participant(s) and the researcher, and whether the researcher was blind to experimental condition and/or the study hypothesis during data collection.*
Timing	*Indicate the start and stop dates of data collection. If there is a gap between collection periods, state the dates for each sample cohort.*
Data exclusions	*If no data were excluded from the analyses, state so OR if data were excluded, provide the exact number of exclusions and the rationale behind them, indicating whether exclusion criteria were pre-established.*
Non-participation	*State how many participants dropped out/declined participation and the reason(s) given OR provide response rate OR state that no participants dropped out/declined participation.*
Randomization	*If participants were not allocated into experimental groups, state so OR describe how participants were allocated to groups, and if allocation was not random, describe how covariates were controlled.*

Ecological, evolutionary & environmental sciences study design

All studies must disclose on these points even when the disclosure is negative.

Study description	*Briefly describe the study. For quantitative data include treatment factors and interactions, design structure (e.g. factorial, nested, hierarchical), nature and number of experimental units and replicates.*
Research sample	*Describe the research sample (e.g. a group of tagged Passer domesticus, all Stenocereus thurberi within Organ Pipe Cactus National Monument), and provide a rationale for the sample choice. When relevant, describe the organism taxa, source, sex, age range and any manipulations. State what population the sample is meant to represent when applicable. For studies involving existing datasets, describe the data and its source.*
Sampling strategy	*Note the sampling procedure. Describe the statistical methods that were used to predetermine sample size OR if no sample-size calculation was performed, describe how sample sizes were chosen and provide a rationale for why these sample sizes are sufficient.*
Data collection	*Describe the data collection procedure, including who recorded the data and how.*
Timing and spatial scale	*Indicate the start and stop dates of data collection, noting the frequency and periodicity of sampling and providing a rationale for these choices. If there is a gap between collection periods, state the dates for each sample cohort. Specify the spatial scale from which the data are taken*
Data exclusions	*If no data were excluded from the analyses, state so OR if data were excluded, describe the exclusions and the rationale behind them, indicating whether exclusion criteria were pre-established.*
Reproducibility	*Describe the measures taken to verify the reproducibility of experimental findings. For each experiment, note whether any attempts to repeat the experiment failed OR state that all attempts to repeat the experiment were successful.*
Randomization	*Describe how samples/organisms/participants were allocated into groups. If allocation was not random, describe how covariates were controlled. If this is not relevant to your study, explain why.*
Blinding	*Describe the extent of blinding used during data acquisition and analysis. If blinding was not possible, describe why OR explain why blinding was not relevant to your study.*

Did the study involve field work? ☐ Yes ☐ No

Field work, collection and transport

Field conditions	*Describe the study conditions for field work, providing relevant parameters (e.g. temperature, rainfall).*
Location	*State the location of the sampling or experiment, providing relevant parameters (e.g. latitude and longitude, elevation, water depth).*
Access & import/export	*Describe the efforts you have made to access habitats and to collect and import/export your samples in a responsible manner and in compliance with local, national and international laws, noting any permits that were obtained (give the name of the issuing authority, the date of issue, and any identifying information).*
Disturbance	*Describe any disturbance caused by the study and how it was minimized.*

Reporting for specific materials, systems and methods

We require information from authors about some types of materials, experimental systems and methods used in many studies. Here, indicate whether each material, system or method listed is relevant to your study. If you are not sure if a list item applies to your research, read the appropriate section before selecting a response.

Materials & experimental systems

n/a	Involved in the study	
☒	☐	Antibodies
☐	☒	Eukaryotic cell lines
☒	☐	Palaeontology and archaeology
☒	☐	Animals and other organisms
☒	☐	Clinical data
☒	☐	Dual use research of concern
☒	☐	Plants

Methods

n/a	Involved in the study	
☒	☐	ChIP-seq
☒	☐	Flow cytometry
☒	☐	MRI-based neuroimaging

Eukaryotic cell lines

Policy information about cell lines and Sex and Gender in Research

Cell line source(s)	The HEK-293T cell line was derived from a female. The cells used in this experiment were obtained from Emory University's Viral Vector Core, which purchased them from Invitrogen.
Authentication	None of the cell lines used were authenticated.
Mycoplasma contamination	Cell lines were tested to verify absence of mycoplasma contamination.
Commonly misidentified lines (See ICLAC register)	The HEK-293T cell line is not listed as a commonly misidentified cell line.

Plants

Seed stocks	*Report on the source of all seed stocks or other plant material used. If applicable, state the seed stock centre and catalogue number. If plant specimens were collected from the field, describe the collection location, date and sampling procedures.*
Novel plant genotypes	*Describe the methods by which all novel plant genotypes were produced. This includes those generated by transgenic approaches, gene editing, chemical/radiation-based mutagenesis and hybridization. For transgenic lines, describe the transformation method, the number of independent lines analyzed and the generation upon which experiments were performed. For gene-edited lines, describe the editor used, the endogenous sequence targeted for editing, the targeting guide RNA sequence (if applicable) and how the editor was applied.*
Authentication	*Describe any authentication procedures for each seed stock used or novel genotype generated. Describe any experiments used to assess the effect of a mutation and, where applicable, how potential secondary effects (e.g. second site T-DNA insertions, mosiacism, off-target gene editing) were examined.*
